# Unravelling the Diagnosis of Normal-Flow Low-Gradient Aortic Stenosis: The Role of Multimodality Imaging

**DOI:** 10.3390/medicina62101849

**Published:** 2026-09-24

**Authors:** Laura Rotondo, Elena Marchetti, Rita Pavasini, Elisabetta Tonet, Carlo Tumscitz, Carlo Penzo, Marta Cocco, Maria Lo Monaco, Erika Bertella, Gianluca Campo, Federico Marchini

**Affiliations:** 1 Cardiology Unit, Azienda Ospedaliero Universitaria di Ferrara, Via Aldo Moro 8, 44124 Ferrara, Italy; laura.rotondo@edu.unife.it (L.R.); elena02.marchetti@edu.unife.it (E.M.); pvsrti@unife.it (R.P.); tonet.elisabetta@gmail.com (E.T.); tumscitz@gmail.com (C.T.); carlopenzo78@gmail.com (C.P.); mcocco92@gmail.com (M.C.); cmpglc@unife.it (G.C.); mrcfrc2@unife.it (F.M.); 2 Cardiovascular Institute, Ospedale Humanitas Gavazzeni, Via Mauro Gavazzeni 21, 24125 Bergamo, Italy; erika.bertella@gavazzeni.it

**Keywords:** aortic stenosis, normal-flow low-gradient, multimodality imaging, transthoracic echocardiography, transoesophageal echocardiography, cardiovascular magnetic resonance, cardiac computed tomography, aortic valve calcium score, invasive hemodynamic assessment

## Abstract

Normal-flow, low-gradient aortic stenosis is one of the most deceptive presentations of aortic stenosis. It leaves clinicians uncertain whether they are facing true severe disease or a measurement artefact. This often reflects a discordant grading pattern, in which many patients ultimately prove to have moderate disease. This diagnostic paradox arises from a combination of measurement errors, geometric assumptions, and genuine hemodynamic complexity, and cannot be resolved by echocardiography alone. A structured multimodality imaging strategy integrating calcium score evaluation on cardiac computed tomography, tissue characterisation on cardiac magnetic resonance and, when necessary, invasive hemodynamic assessment is the only reliable path to diagnostic certainty and appropriate therapeutic decision-making. Together, these modalities form a coherent sequential algorithm that moves from the non-invasive to the invasive, from the hemodynamic to the biological, and from uncertainty to actionable clinical decision. This review provides an updated, critical appraisal of each imaging tool and its respective strengths and pitfalls.

## 1. Introduction

### 1.1. Definition and Epidemiology

Aortic stenosis (AS) is the most prevalent valvular heart disease in developed countries, with a prevalence of approximately 1–3% in adults over 65 years of age and a significant increase in incidence with advancing age [1,2]. In its classic hemodynamic presentation, severe AS is characterised by an aortic valve area (AVA) ≤ 1.0 cm^2^, a mean transvalvular gradient ≥ 40 mmHg, and a peak aortic jet velocity ≥ 4 m/s. However, a substantial proportion of patients, estimated between 15% and 40% of those with echocardiographically graded severe AS, present with a discordant hemodynamic pattern, in which a reduced AVA consistent with severe stenosis coexists with a mean transvalvular gradient below 40 mmHg [3,4]. This condition, broadly referred to as low-gradient aortic stenosis (LG-AS), encompasses distinct pathophysiological entities that require careful differentiation to guide clinical decision-making.

### 1.2. Pathophysiology and Diagnostic Challenges

Contemporary classification distinguishes three main subtypes of LG-AS. Classical low-flow, low-gradient AS (LF-LG AS) is defined by a reduced left ventricular ejection fraction (LVEF < 50%) and a stroke volume index (SVi) ≤ 35 mL/m^2^, reflecting impaired systolic function as the primary driver of the low gradient [3]. Paradoxical low-flow, low-gradient AS is characterised by preserved LVEF (≥50%) but a reduced SVi (≤35 mL/m^2^), resulting from a small, hypertrophied, and restrictive left ventricle operating at reduced stroke volume despite normal ejection fraction [5]. The third subtype is normal-flow, low-gradient AS (NF-LG AS), defined by AVA ≤1.0 cm^2^, mean gradient < 40 mmHg, preserved LVEF (≥50%), and a normal SVi (>35 mL/m^2^) [3,6]. This entity is particularly challenging because the low gradient cannot be attributed to reduced transvalvular flow, raising the fundamental question of whether the AVA measurement truly reflects severe stenosis or whether it represents a measurement artefact.

Several mechanisms may account for the discordance between AVA and gradient in NF-LG AS. These include technical errors, such as mean gradient underestimation due to suboptimal Doppler alignment, and underestimation of the LVOT area caused by assuming a circular geometry from a single antero-posterior diameter, whereas the LVOT is physiologically elliptical. Additionally, failing to index AVA to body surface area can lead to misclassification in smaller patients. Additionally, this discordance can be explained by the intrinsic flow-dependence of the transvalvular gradient, which may remain low despite anatomically severe leaflet obstruction in patients with reduced systemic arterial compliance, concentric hypertrophy, reduced ventricular compliance, and small cavity size [3,4,7,8]. In a subset of these patients, these hemodynamic features reflect true severe stenosis rather than measurement artefact, and are associated with a worse prognosis than truly moderate AS [9,10]. Even though identifying truly severe AS remains essential, multimodality reclassification studies showed that most of these patients have moderate AS: in fact, hybrid MDCT-Doppler fusion reclassifies 35–52% of NF-LG AS cases from severe to moderate [11,12].

### 1.3. Rationale of Multimodality Imaging

The accurate identification of true severe AS in the NF-LG subset is clinically critical, as aortic valve replacement (AVR), whether surgical (SAVR) or transcatheter (TAVI/TAVR), has been shown to improve outcomes in patients with confirmed severe disease, while exposing patients with pseudo-severe or moderate AS to unnecessary procedural risk [6,10]. The most recent 2025 ESC/EACTS Guidelines for the Management of Valvular Heart Disease and the 2020 ACC/AHA Guideline for the Management of Patients with Valvular Heart Disease recognise NF-LG AS as a diagnostic challenge requiring multimodality imaging for confirmation of stenosis severity [6,13]. Notably, the 2025 ESC/EACTS guidelines formally operationalize multimodality adjudication as a mandatory step in discordant AS, embedding it within a structured diagnostic algorithm and acknowledging that discordant grading is common and cannot be resolved by echocardiography alone [6,14].

A comprehensive multimodality imaging approach integrating transthoracic and transoesophageal echocardiography (TTE/TOE), stress echocardiography, cardiac magnetic resonance (CMR), multidetector computed tomography (MDCT), and, in selected cases, invasive hemodynamic assessment is therefore essential to resolve diagnostic uncertainty, characterise the extent of myocardial remodelling, and guide therapeutic planning (Figure 1, Table 1). This review provides an updated, structured appraisal of each imaging modality in the assessment of NF-LG AS, discussing its respective contributions, limitations, and complementary roles within an integrated diagnostic algorithm.

## 2. Echocardiography

Echocardiography represents the gold standard for the initial evaluation in patients with suspected severe AS [6,15]; however, its clinical reliability is challenged by the NF-LG phenotype due to the diagnostic mismatch on standard Doppler evaluation.

### 2.1. Transthoracic Echocardiography

When an NF-LG AS is identified on standard TTE, the primary clinical objective is to differentiate true severe AS from a false-positive presentation caused by measurement errors or systemic factors. AVA calculation, via the continuity equation, is widely used as a “less flow-dependent” parameter that can be employed to assess AS severity even in low-flow states. However, this calculation relies on several operator-dependent steps and thus small inaccuracies can have a significant impact on the final diagnosis.

The most common source of error in TTE is the linear measurement of the LVOT diameter in the parasternal long-axis view (Figure 2) since the continuity equation squares this diameter to calculate the cross-sectional area, assuming a perfectly circular geometry. However, because the human LVOT is frequently elliptical, 2D-TTE systematically underestimates its true area [16]. This systematic error artificially reduces the calculated stroke volume and AVA, creating a false-positive severe AS profile in a patient who may only have moderate stenosis. Evaluation of the velocity time integral ratio (VR), also referred to as dimensionless velocity index (DVI), can circumvent this limitation. In fact, by providing a ratio of the VTI at the aortic valve and at the LVOT, DVI avoids operator-dependent measurement errors of the LVOT. Yet, in the setting of NF-LG AS, DVI also carries some limitations. In a recent large multicenter study by Altes et al., the authors demonstrated that a significantly reduced DVI (less than 0.25) carries a powerful, independent prognostic value for all-cause mortality in LG AS, but also that this risk-stratification capacity is highly flow-dependent. Specifically, while a ratio less than 0.25 strongly predicted poor long-term outcomes in patients with low-flow states, it did not retain the same incremental prognostic weight within the normal-flow subgroup. In patients presenting with a true NF-LG pattern, survival rates remained favourable and closely aligned with those observed in moderate AS, regardless of whether the DVI was above or below the 0.25 threshold [17]. These findings suggest that a low DVI in an NF-LG patient should not be taken as definitive proof of clinical severity, but rather as an indication for further multi-parametric confirmation to differentiate true severe stenosis from a more benign phenotype.

Another common source of error during TTE is represented by Doppler Beam Misalignment. Accurate gradient assessment requires strict parallel alignment between the continuous-wave (CW) Doppler beam and the high-velocity aortic jet. Suboptimal alignment or an inadequate acoustic window results in an underestimation of both peak velocity and mean pressure gradient. Consequently, a true high-gradient severe AS can be mistakenly categorised as an NF-LG phenotype due to technical limitations. Apical windows are usually preferred for aortic jet velocity measurements; however, according to the literature, apical windows identify the maximum velocity in only 40% of patients, whereas the maximum velocity is obtained from the right parasternal windows in up to 50% of patients [18].

Systemic hypertension can also alter valvular evaluation. In fact, hypertension alters the ventriculo-arterial coupling and increases the left ventricular afterload. An elevated blood pressure during examination increases afterload, reducing transvalvular flow, pressure gradients, and stroke volume, which in turn leads to an underestimation of the calculated AVA via the continuity equation. This transient hemodynamic state can mimic an NF-LG pattern. Consequently, blood pressure must be evaluated before confirming diagnosis by TTE, and hypertensive patients should be re-examined following blood pressure control.

In order to better categorise the flow state in NF-LG AS, transvalvular flow rate (FR) has been proposed as an alternative to stroke volume [19]. Transvalvular FR is defined as the SV divided by the systolic ejection time and represents the volume of fluid that passes across the valve per unit of time. Notably, a normal SVi does not guarantee a normal FR. In fact, flow-volume discordance has been found in nearly one third of patients with AS who underwent TAVI and offers superior prognostic value over existing SVi and FR thresholds [20]. Moreover, a significant number of NF-LG patients exhibit a reduced FR despite a normal SV, with a cutoff of 210 mL/s commonly used to define a reduced value, a threshold that has also been shown to retain independent prognostic value [21]. This phenomenon is especially observed in elderly individuals with pronounced arterial stiffness or bradycardia [22,23]. In these patients the LV faces an increased afterload with a consequent prolongation of the ejection time, resulting in a lower instantaneous velocity and pressure gradient.

Finally, current ESC guidelines endorse the use of indexed Aortic Valve Area (AVAi), defining a severity threshold of <0.6 cm^2^/m^2^. Evaluation of AVAi is primarily important to identify the “small body size” phenotype. In constitutionally small patients (typically presenting with a Body Surface Area, BSA < 1.6 m^2^), an AVA < 1.0 cm^2^ may falsely suggest severe stenosis. In this setting, the presence of an AVAi ≥ 0.6 cm^2^/m^2^ effectively reclassifies the condition as moderate. Conversely, in patients with a Body Mass Index (BMI) ≥ 30 kg/m^2^, there is an expansion of the calculated BSA without a proportional increase in transvalvular metabolic blood flow requirements. This geometric distortion could drive the AVAi below the 0.6 cm^2^/m^2^ threshold, leading to a systematic overestimation of stenosis severity. Consequently, in obese patients, diagnosis should rely on the non-indexed AVA threshold of 1.0 cm^2^ [24].

Stress echocardiography (SE) with low-dose dobutamine is used in classical low-flow, low-gradient AS to distinguish true severe from pseudo-severe stenosis, using the projected aortic valve area at a standardised flow rate rather than the peak-stress gradient alone, since stress-induced flow can raise the gradient above 40 mmHg even in anatomically moderate AS [25,26]. In genuine NF-LG AS, where resting SVi and flow rate are already normal, stress echocardiography generally adds limited diagnostic value. Patients with a normal SVi but a borderline flow rate, reflecting prolonged ejection time, arterial stiffness, or bradycardia, may still derive incremental diagnostic value from flow-rate-based stress testing.

### 2.2. Transoesophageal Echocardiography

TOE has a fundamental role in the setting of AS when transthoracic acoustic windows are suboptimal or when discordant hemodynamic parameters persist.

Thanks to its spatial resolution and proximity to the aortic valve complex, TOE allows for direct anatomical assessment of the aortic valve apparatus and evaluation of the planimetric area. In the past, direct assessment of the anatomical orifice via 2D TOE planimetry was proposed to bypass the fluid-dynamic assumptions of the continuity equation. However, conventional 2D planimetry carries severe methodological limitations, including the risk of foreshortening artefact. The introduction of 3D multiplanar reconstruction (MPR) has substantially addressed the limitations of both the 2D continuity equation and 2D planimetry. In fact, the clinician can manipulate the dataset in the X, Y, and Z axes simultaneously. This precise alignment ensures that the short-axis cutting plane is positioned exactly at the smallest anatomical opening at the very tips of the calcified leaflets during peak mid-systole. A 3D-MPR planimetric AVA < 1.0 cm^2^ can provide anatomical support for severe AS. However, direct planimetry evaluation has several limitations, especially in heavily calcified valves.

In addition, TOE can also increase the accuracy of calculated AVA by better evaluation of the LVOT area. As previously noted, standard 2D-TTE assumes a perfectly circular LVOT, measuring only the anteroposterior diameter in the parasternal long-axis view. MPR via 3D-TOE allows for direct tracing of the actual, often elliptical, cross-sectional area of the LVOT [16,27,28]. Incorporating the true 3D-measured LVOT area into the continuity equation prevents the artificial underestimation of SV seen with 2D-based measurements. This methodological correction frequently allows for reclassification of NF-LG patients into their true pathophysiological categories.

## 3. Computed Tomography

### 3.1. Aortic Valve Calcium Scoring

MDCT plays a pivotal and increasingly central role in the evaluation of NF-LG AS, providing anatomical and structural information that is fundamentally independent of transvalvular flow and therefore immune to the hemodynamic variability that limits standard Doppler echocardiography. The cornerstone CT-based parameter in this context is the aortic valve calcium score (AVCS), quantified using the Agatston method on non-contrast, electrocardiogram-gated images (Figure 3) [29,30].

The pathophysiological rationale for using the AVCS in discordant AS is straightforward: the degree of valvular calcification directly reflects the anatomical burden of stenosis and correlates with the hemodynamic severity of the disease independently of flow state [7]. In patients with NF-LG AS, where both elevated afterload and measurement artefacts may simultaneously contribute to discordance, an objectively quantified anatomical parameter provides a critical anchor for clinical decision-making. Clavel et al. first proposed sex-specific AVCS thresholds to diagnose severe AS (1274 AU in women and 2065 AU in men) derived from a cohort with concordant echocardiographic grading, preserved LVEF, and normal flow [30]. These thresholds were subsequently validated in larger cohorts and progressively refined, forming the basis of current guideline recommendations.

It should be noted that the validated AVCS thresholds for the NF-LG subtype were largely derived from populations with normal flow and preserved or mildly reduced LVEF, conditions that correspond well to NF-LG AS, making them more directly applicable here than in the classical LF-LG or paradoxical LF-LG subtypes.

A clinically relevant consideration is the well-documented sex difference in AVCS accumulation for a given hemodynamic severity of AS. It has been observed that women consistently exhibit severe stenosis with lower AVCS in comparison to men. This phenomenon is likely indicative of a greater contribution of fibrotic leaflet thickening, rather than calcification per se, to the restriction of systolic valve opening. This sex difference in pathogenesis, with more fibrosis and less calcification in women, may be even more accentuated with increasing severity of AS, which explains the lower sex-specific threshold applied to women and underscores the importance of avoiding a single universal cut-off. The AVCS density (AVCS normalised to the annular area, expressed in AU/cm^2^) has been proposed as a complementary parameter that partially corrects for annulus size variability across patients, though its incremental value over the standard Agatston score remains debated [31].

Both the 2025 ESC/EACTS and the 2020 ACC/AHA guidelines endorse AVCS as a Class IIa recommendation to adjudicate severity in patients with discordant or low-gradient AS, including the NF-LG subtype, though they differ in the specific thresholds applied. The 2025 ESC/EACTS guidelines incorporate CT aortic valve calcium scoring with sex-specific thresholds of >1200 AU in women and >2000 AU in men as part of a formal multimodality algorithm for discordant AS, formally acknowledging that such adjudication is no longer optional in high-stakes clinical decisions. The ACC/AHA 2020 guidelines adopt numerically similar but not identical binary cut-offs, and the two documents diverge in how intermediate values are interpreted [6,13]. These thresholds are best applied as part of an integrated, probabilistic assessment rather than as rigid cut-offs, especially at intermediate values, and may perform less well in younger patients, bicuspid valves, or predominantly fibrotic rather than calcific phenotypes.

An important caveat concerns patients with concomitant transthyretin cardiac amyloid (ATTR-CA), whose prevalence in elderly patients undergoing TAVI ranges from 4% to 16% and increases progressively with age [32,33]. In this subgroup, the calcium score may not reliably reflect the true hemodynamic severity of AS. In the CAUSATIVE study, Serenelli et al. demonstrated that patients with severe AS and coexistent cardiac amyloidosis had significantly lower aortic valve calcium scores compared to AS patients without amyloidosis, despite equivalent hemodynamic severity. This suggests that factors beyond leaflet calcification may contribute to hemodynamic severity in this subgroup, although the underlying mechanism remains unproven. Calcium score thresholds validated in the general AS population may therefore underrepresent true severity specifically in patients with ATTR-CA, reinforcing the value of a multimodality approach and of active ATTR-CA screening in this subgroup [34]. Moreover, MDCT is increasingly recognised as a platform for active myocardial tissue characterisation. Extracellular volume quantification by CT (ECV-CT), derived by comparing myocardial and blood pool attenuation in Hounsfield Units between non-contrast and delayed post-contrast acquisitions, corrected for haematocrit, represents an emerging technique for non-invasive assessment of myocardial interstitial expansion. ECV-CT is markedly elevated in the presence of ATTR-CA and has been validated as a discriminator between AS patients with and without concomitant cardiac amyloidosis, demonstrating high sensitivity and negative predictive value for excluding significant scintigraphic uptake, supporting its potential role as a gatekeeper for targeted downstream screening [35,36]. However, ECV-CT requires a dedicated delayed acquisition not routinely performed and lacks universally validated thresholds due to significant protocol variability across studies. These developments position MDCT as an evolving platform for myocardial tissue characterisation in the AS population, though broader clinical implementation awaits protocol standardisation and prospective validation.

### 3.2. LVOT Area Measurement and Hybrid AVA Calculation

Beyond calcium scoring, MDCT provides a direct and geometrically accurate measurement of the LVOT cross-sectional area, overcoming the limitations associated with the assumption of a circular LVOT geometry when applying the continuity equation in two-dimensional echocardiography [37].

MDCT planimetry of the LVOT allows precise measurement of both the major and minor diameters of its elliptical cross-section, enabling accurate area computation. This CT-derived LVOT area can then be combined with the Doppler velocity-time integral from transthoracic echocardiography in a so-called hybrid AVA approach. In a study by Kamperidis et al. including 191 patients with severe AS and preserved LVEF, the use of hybrid MDCT-Doppler fusion AVAi reclassified 52% of patients with NF-LG AS and 12% of those with LF-LG AS into true moderate AS, by providing the true cross-sectional LVOT area through MDCT planimetry. This finding has significant clinical implications: it suggests that a substantial proportion of patients currently labelled as NF-LG severe AS may in fact harbour moderate stenosis and be spared unnecessary procedural intervention [11].

The clinical utility of the hybrid AVA approach remains debated. In a retrospective study of 359 consecutive patients undergoing TAVI for low-gradient severe AS, 35% were reclassified to moderate AS by fusion imaging; however, clinical outcome at one year (defined as the composite of all-cause mortality and rehospitalization for heart failure) was similar between patients reclassified to moderate or retained as severe AS, suggesting that reclassification by hybrid AVA does not translate into a prognostic benefit [12]. Furthermore, direct planimetry of the AVA by MDCT, measuring the valve orifice area directly at the leaflet tips, has shown limited diagnostic performance compared to the hybrid method, with CT-derived direct AVA cut points substantially higher than those used for echocardiographic AVA to achieve adequate specificity for excluding severe AS, highlighting the technical challenges of identifying the true minimal orifice area in a heavily calcified, irregularly shaped valve [38]. Despite these limitations, MDCT-based LVOT measurement remains a valuable tool for correcting systematic underestimation by echocardiography in selected cases.

### 3.3. TAVI Planning and Anatomical Assessment

In patients with NF-LG AS in whom TAVI is selected as the therapeutic strategy, MDCT assumes a mandatory pre-procedural role that extends well beyond severity assessment. Electrocardiogram-gated contrast-enhanced CT of the aortic root, aorta, and peripheral vasculature provides the anatomical information required for annular sizing, device selection, vascular access planning, and risk assessment for procedural complications including coronary obstruction, annular rupture, and paravalvular leak [39]. Coronary height, leaflet calcification distribution, and ilio-femoral vessel characteristics are systematically evaluated to determine transfemoral access feasibility and estimate procedural risk [40].

A clinically relevant novelty introduced by the 2025 ESC/EACTS guidelines concerns the coronary work-up in TAVI candidates. In patients with a low or moderate pre-test likelihood of obstructive coronary artery disease (≤50%), coronary CT angiography (CCTA) is now recommended (with a Class I, Level B indication) to rule out significant CAD prior to valve intervention, given its high sensitivity of 95–97% for the detection of obstructive disease in this population [6]. This represents a major paradigm shift toward a CT-first approach in pre-intervention coronary assessment, limiting invasive coronary angiography to patients with higher pre-test probability or specific clinical conditions. In practical terms, CCTA can be integrated into the same acquisition session as the structural TAVI planning CT and the non-contrast AVC scoring sequence, consolidating the entire pre-procedural imaging workup (severity adjudication, procedural planning, and coronary assessment) within a single CT study. Finally, a clinically relevant but often overlooked contribution of pre-TAVI MDCT is the detection of incidental extracardiac findings. Given the large anatomical coverage of the acquisition, spanning from the thoracic inlet to the femoral arteries, a substantial proportion of TAVI candidates are found to harbour unexpected findings of potential clinical significance, including pulmonary nodules, aortic aneurysms, and solid organ lesions. Recent evidence suggests that such incidental findings are common in this population; however, they do not appear to adversely affect procedural outcomes or overall prognosis and should not delay or preclude valve intervention in patients with confirmed severe AS [41].

## 4. Cardiac Magnetic Resonance

### 4.1. AVA Assessment and Flow Quantification

CMR imaging occupies a unique position in the multimodality evaluation of NF-LG AS, offering complementary capabilities with respect to both echocardiography and CT. Its principal contributions span two distinct but interconnected domains: hemodynamic assessment of AS severity through accurate flow quantification and AVA calculation and myocardial tissue characterisation, which represents the most distinctive and clinically impactful strength of this modality. Although CMR is not routinely performed in all patients with valvular heart disease, it provides useful information encompassing ventricular volume and function, tissue characterisation, and flow quantification, while avoiding the main limitations of other non-invasive modalities [42,43].

Hemodynamic assessment by CMR relies primarily on two-dimensional phase-contrast velocity encoding (PC-CMR), which enables direct measurement of blood velocity and volumetric flow across both the aortic valve and the LVOT. The continuity equation can then be applied to PC-CMR-derived flow data to calculate AVA without assuming LVOT circularity, since CMR cine imaging allows direct planimetry of the true elliptical LVOT cross-sectional area [43]. In a validation study of 50 patients with moderate or severe AS who underwent TTE, cardiac catheterization, and CMR, PC-CMR-derived AVA showed excellent intra- and interobserver reproducibility, with intraclass correlation coefficients of 0.939 and 0.827, respectively, and a small bias compared to invasive AVA determination by the Gorlin formula comparable to that observed with standard TTE. These characteristics make PC-CMR a valuable second-line tool for severity confirmation when echocardiographic data are technically suboptimal or internally inconsistent [44].

A recognised limitation of PC-CMR in severe AS is signal dephasing due to turbulent, high-velocity transvalvular flow, which can result in underestimation of peak velocities and derived hemodynamic parameters. The accuracy of PC-CMR for stroke volume measurement may be altered in severe AS by turbulent flow-related intravoxel dephasing, and the choice of imaging plane significantly affects the magnitude of this error, with LVOT-level acquisitions showing greater susceptibility in high-gradient disease. Optimising the echo time and acquisition plane selection mitigates this effect and improves the reliability of PC-CMR in the most severe stenoses [44,45].

An additional limitation of 2D PC-CMR is its strict dependence on prospective acquisition plane planning, as it requires the imaging slice to be positioned precisely perpendicular to the direction of transvalvular flow at the time of acquisition. Any off-axis angulation relative to the true flow direction, which may be difficult to predict from pre-scan anatomical images, particularly in the presence of eccentric or complex stenotic jets as commonly encountered in calcific AS, introduces systematic underestimation of velocities and derived flow volumes. This constraint renders 2D PC-CMR intrinsically operator-dependent and potentially unreliable in cases with complex flow profiles, constituting a fundamental limitation [44,45].

Direct planimetric measurement of AVA from cine CMR images in the short-axis plane of the aortic valve at peak systole is also feasible and has shown strong correlation with TOE planimetry in patients without extensive leaflet calcification; however, its accuracy deteriorates markedly in the presence of heavy calcification, which causes signal loss and obscures leaflet edges, limiting its applicability in the calcific AS population that characterises NF-LG AS [43].

Stress CMR with low-dose dobutamine has been investigated as an alternative to stress echocardiography for the assessment of LV flow reserve and the distinction between true severe and pseudo-severe AS in the low-flow, low-gradient subtypes, and has been shown to be feasible and safe even in patients with moderate-to-severe AS [42,46]. However, in NF-LG AS, where stroke volume index is by definition preserved, the hemodynamic rationale for provocative flow augmentation does not apply, and stress CMR has no established diagnostic role in this specific subtype.

### 4.2. Myocardial Tissue Characterisation: LGE, T1 Mapping, and ECV

The most clinically distinctive contribution of CMR in NF-LG AS lies in its unparalleled capacity for non-invasive myocardial tissue characterisation. Chronic pressure overload imposed by AS triggers a cascade of maladaptive responses such as cardiomyocyte hypertrophy, interstitial fibrosis, and ultimately replacement fibrosis, that progress in parallel with hemodynamic severity and contribute independently to symptoms, LV dysfunction, and long-term outcomes [42,43].

Late Gadolinium Enhancement (LGE) identifies focal replacement fibrosis through the differential washout kinetics of gadolinium-based contrast agents. In AS, the predominant pattern is mid-wall linear enhancement in the interventricular septum, reflecting non-ischemic pressure-overload fibrosis distinct from ischemic subendocardial or transmural patterns [43,47]. In a systematic review and meta-analysis of six studies comprising 1151 patients with AS, LGE was present in 49.1% of patients and was a strong predictor of all-cause mortality both on univariate analysis and after adjustment for baseline characteristics, with no significant heterogeneity across studies. Importantly, the presence of LGE was also independently associated with all-cause mortality in patients with severe AS following aortic valve replacement, suggesting that pre-existing replacement fibrosis limits the functional recovery achievable after successful valve intervention [47].

However, LGE has an intrinsic limitation: it detects only focal, advanced fibrosis and is insensitive to the earlier and more diffuse pattern of interstitial fibrosis that characterises the initial phases of pressure-overload myocardial remodelling, a distinction particularly relevant in NF-LG AS, where patients may present at a relatively earlier stage of myocardial injury [47,48]. T1 mapping and ECV address precisely this gap, enabling quantification of diffuse myocardial fibrosis without the threshold effect inherent to LGE [48]. ECV, derived from pre- and post-contrast T1 measurements corrected for haematocrit, provides a direct estimate of the fractional volume of the extracellular compartment and is the most validated surrogate for diffuse interstitial fibrosis currently available [48]. In a large international multicenter study of 440 patients with severe AS from 10 centres who underwent CMR with T1 mapping before aortic valve intervention, ECV correlated with markers of left ventricular decompensation including LV mass, left atrial volume, NYHA class III/IV, presence of LGE, and lower LVEF; after a median follow-up of 3.8 years, a progressive increase in all-cause mortality was observed across ECV tertiles (17.3, 31.6, and 52.7 deaths per 1000 patient-years), and ECV was independently associated with all-cause mortality after adjustment for established prognostic markers [49].

In the specific context of NF-LG AS, tissue characterisation by CMR carries clinical relevance: patients in this subtype present with preserved LVEF and normal flow, parameters that do not capture subclinical myocardial injury. ECV and LGE provide biological complementary information to hemodynamics, potentially identifying patients with advanced myocardial remodelling who may benefit from earlier intervention, although prospective data specifically addressing CMR-guided intervention timing in NF-LG AS remain limited.

A further clinically relevant application of CMR tissue characterisation in the AS population is the non-invasive detection of ATTR-CA. As discussed in the CT section, the coexistence of ATTR-CA alters the calcification pattern of the aortic valve and renders calcium score thresholds unreliable for severity adjudication [34]. Beyond this diagnostic limitation, ATTR-CA carries independent prognostic implications that fundamentally alter the patient’s therapeutic trajectory, given the availability of effective disease-modifying therapies. Elevated native T1 and ECV on CMR are early and sensitive markers of amyloid infiltration, detectable even before LGE becomes positive, and an unexpectedly high ECV or a disproportionate degree of myocardial hypertrophy relative to hemodynamic severity should prompt consideration of dual pathology [32,33]. In this context, CMR tissue characterisation serves not only as a prognostic tool for AS itself, but as a screening platform for a treatable comorbidity whose recognition may substantially modify clinical management.

### 4.3. Four-Dimensional Flow MRI

Four-dimensional flow MRI (Four-dimensional flow MRI), a time-resolved three-dimensional phase-contrast imaging technique, represents the most advanced CMR application in aortic valve disease. Four-dimensional flow MRI offers the opportunity to derive advanced hemodynamic measures including vorticity and helicity, wall shear stress, flow displacement, pressure gradients, viscous energy loss, and turbulent kinetic energy, providing comprehensive visualisation and quantification of in vivo aortic flow pattern dynamics in the setting of aortic valve disease. A fundamental technical advantage that distinguishes 4D flow MRI from conventional 2D PC-CMR is its fully three-dimensional, time-resolved acquisition: whereas 2D PC-CMR encodes velocity data in a single pre-planned slice, 4D flow MRI acquires velocity information throughout an entire cardiac volume, allowing flow to be interrogated along any plane within that volume without the need for repeat acquisition. 4D flow MRI thus overcomes the spatial orientation constraints of 2D PC-CMR, offering a more robust framework for transvalvular flow assessment in complex anatomical settings such as NF-LG AS. These parameters capture the mechanistic link between transvalvular flow disturbance and downstream myocardial and aortic wall remodelling, a dimension of disease biology invisible to conventional imaging. From a practical standpoint, 4D flow MRI currently remains primarily a research tool, limited by long acquisition times, complex post-processing requirements, and the absence of standardised reference values across vendors and field strengths; its clinical application in NF-LG AS specifically has not yet been systematically evaluated [50].

## 5. Invasive Hemodynamic Assessment

Cardiac catheterization represents the final diagnostic resource in patients with NF-LG AS in whom the aggregate of non-invasive imaging fails to resolve the discordance between AVA and gradient. Its role is not systematic but selective, reserved for cases where the clinical decision cannot be safely grounded on non-invasive data alone [6,51].

Invasive assessment offers a direct, angle-independent measurement of the mean transvalvular pressure gradient through simultaneous left ventricular and ascending aortic pressure recording, from which AVA is calculated via the Gorlin formula. Accurate simultaneous pressure acquisition using dual-lumen catheters or a combined catheter and pressure wire technique is essential, as pullback tracings and peak-to-peak gradients do not reflect true physiological pressure differences and should not be used as the primary measure of severity [51,52]. Cardiac output measurement, required by the Gorlin formula, introduces an additional source of variability: thermodilution may be inaccurate in patients with low output or tricuspid regurgitation, while the Fick method relies on oxygen consumption values that are rarely measured directly and are instead estimated from age- and sex-based formulas, a practice that introduces non-negligible error in the elderly AS population [51,52].

Beyond these technical considerations, the Gorlin formula carries an intrinsic limitation that is particularly relevant in the low-gradient context. The formula is flow-dependent, and in low-flow states the severity of AS may be overestimated, as the weakly contracting ventricle produces insufficient leaflet opening of a moderately diseased valve, a condition known as pseudo-aortic stenosis. In NF-LG AS, where flow is by definition preserved, this limitation is less pronounced than in the classical LF-LG subtype; nonetheless, beat-to-beat variability in cardiac output and gradient, compounded by the impact of atrial fibrillation on averaged hemodynamic measurements, means that the Gorlin-derived AVA carries uncertainty comparable to that of echocardiographic estimates [51,52]. Furthermore, invasive hemodynamic assessment in patients with low-gradient AS and preserved ejection fraction commonly results in discordant grading in a substantial proportion of patients, with AVA and gradient still pulling in opposite directions, confirming that catheterization does not eliminate the diagnostic uncertainty inherent to this subtype but reframes it with a different set of measurement assumptions. An additional source of discrepancy between invasive and Doppler-derived gradients is the pressure recovery phenomenon: the partial reconversion of kinetic energy to static pressure downstream from the stenotic jet results in a lower gradient measured at the catheter tip compared to the Doppler-derived gradient, particularly in patients with a small ascending aorta [51,52].

## 6. Conclusions and Future Perspectives

Normal-flow, low-gradient aortic stenosis is a diagnostically challenging entity that resists resolution by a single imaging modality and demands a structured multimodality approach. The evidence reviewed supports a sequential algorithm in which echocardiographic optimisation represents the mandatory first step, followed by MDCT aortic valve calcium scoring, the most validated and flow-independent tool for severity adjudication, as the primary second-line investigation. Cardiac MRI is reserved for cases with intermediate calcium scores or diagnostic uncertainty, contributing accurate flow quantification and tissue characterisation with non-invasive quantification of fibrotic remodelling through LGE, T1 mapping, and ECV. Invasive hemodynamic assessment remains the final arbiter in the most challenging cases, with the awareness that catheterization reframes rather than resolves the discordance inherent to this subtype. This stepwise sequence is a proposed practical algorithm, grounded in the general principles of the 2025 ESC/EACTS and 2020 ACC/AHA guidelines, both of which mandate multimodality adjudication in discordant AS though neither prescribes this exact order [6,13].

Several important questions remain unanswered. The most clinically pressing question is the optimal timing of AVR in confirmed NF-LG severe AS. Observational data, drawn largely from broader preserved-EF low-gradient cohorts, suggest a survival benefit from AVR in symptomatic patients. However, these cohorts are heterogeneous and not directly comparable to NF-LG AS. The first dedicated randomised evidence, the ROTAS trial, was limited by small sample size and population heterogeneity, so larger NF-LG-specific trials are warranted [53,54]. Moreover, the role of CMR tissue characterisation as a trigger for earlier intervention is still a matter of debate. However, the first randomised trial to test it (EVOLVED) showed no significant reduction in death or AS-related hospitalisation with early intervention [55].

Looking ahead, several emerging tools hold promise for refining the diagnostic and prognostic assessment of NF-LG AS. ^18^F-NaF PET imaging of active valvular calcification may provide dynamic information on disease progression not captured by static calcium scoring [56,57]. Artificial intelligence applied to multimodality imaging datasets could integrate the multidimensional information generated by echo, CT, and CMR into unified diagnostic classifiers, reducing inter-operator variability. The recognition of concomitant ATTR-CA in up to 16% of elderly TAVI candidates further positions CMR tissue characterisation as a screening platform for a treatable comorbidity that independently worsens prognosis [32,33]. Finally, 4D flow MRI, still primarily a research tool, may in the future provide novel hemodynamic biomarkers of severity and progression complementary to current anatomical and tissue parameters [50].

In conclusion, the multimodality imaging evaluation of NF-LG AS has matured considerably over the past decade, evolving from an echocardiography-centric approach into a structured, biology-informed algorithm. The coming years will likely see this algorithm enriched by molecular imaging, AI-assisted data integration, and prospective randomised evidence on intervention timing, moving the field toward a truly individualised approach to one of the most prevalent and clinically demanding valvular conditions in the ageing population.

## Figures and Tables

**Figure 1:**
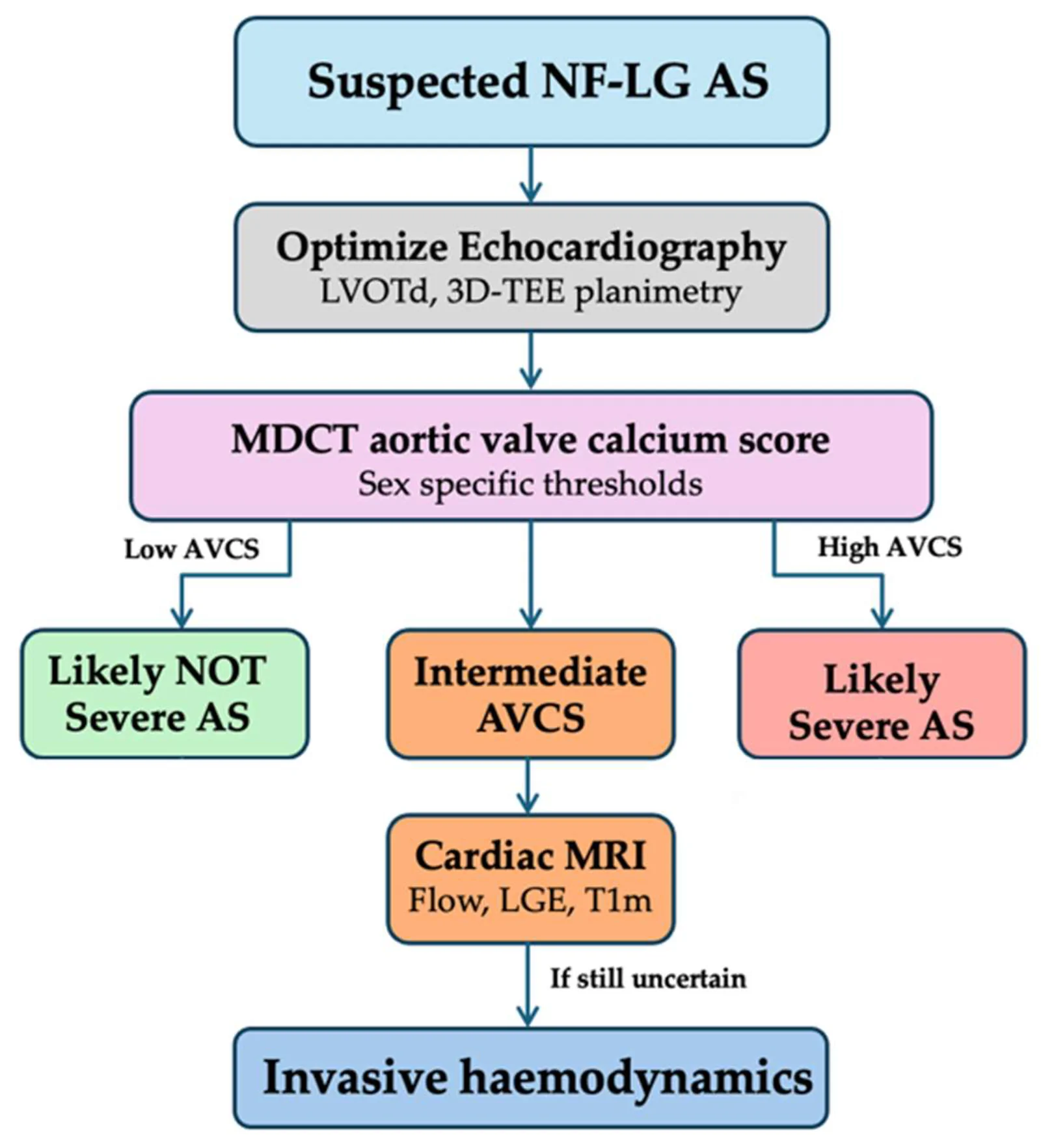
A practical example of diagnostic flowchart in NF-LG AS. AS, aortic stenosis; NF-LG, normal-flow, low-gradient; LVOT, left ventricle outflow tract; TOE, transoesophageal; MDCT, multidetector computed tomography; AVCS, aortic valve calcium score; MRI, magnetic resonance.

**Figure 2:**
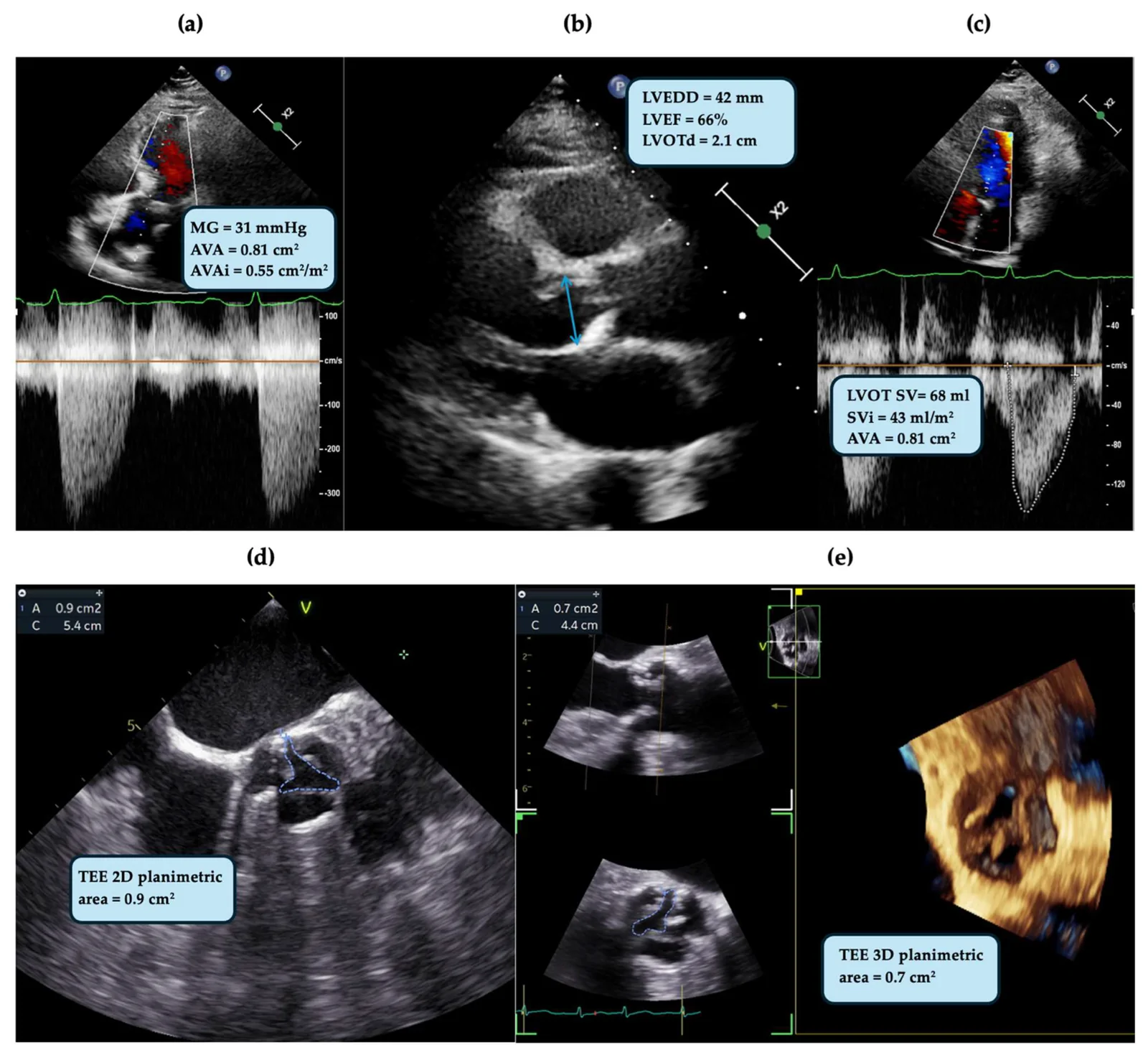
Patient with a Normal-Flow, Low-Gradient Aortic Stenosis. Case of a 63-year-old man with symptomatic dyspnoea, NYHA class III. The patient presented with normal LVEF and normal transvalvular flow (**a**,**b**). AVA calculation revealed likely severe stenosis with low mean transvalvular gradient (31 mmHg) (**a–c**). Subsequent TOE evaluation confirmed the presence of severe stenosis in both 2D and 3D planimetric AVA evaluations (**d**,**e**). MG, mean gradient; AVA, aortic valve area; LVEDD, left ventricle diastolic diameter; LVEF, left ventricle ejection fraction; LVOTd, left ventricle outflow tract diameter; SV, stroke volume; TOE, transoesophageal echocardiography.

**Figure 3:**
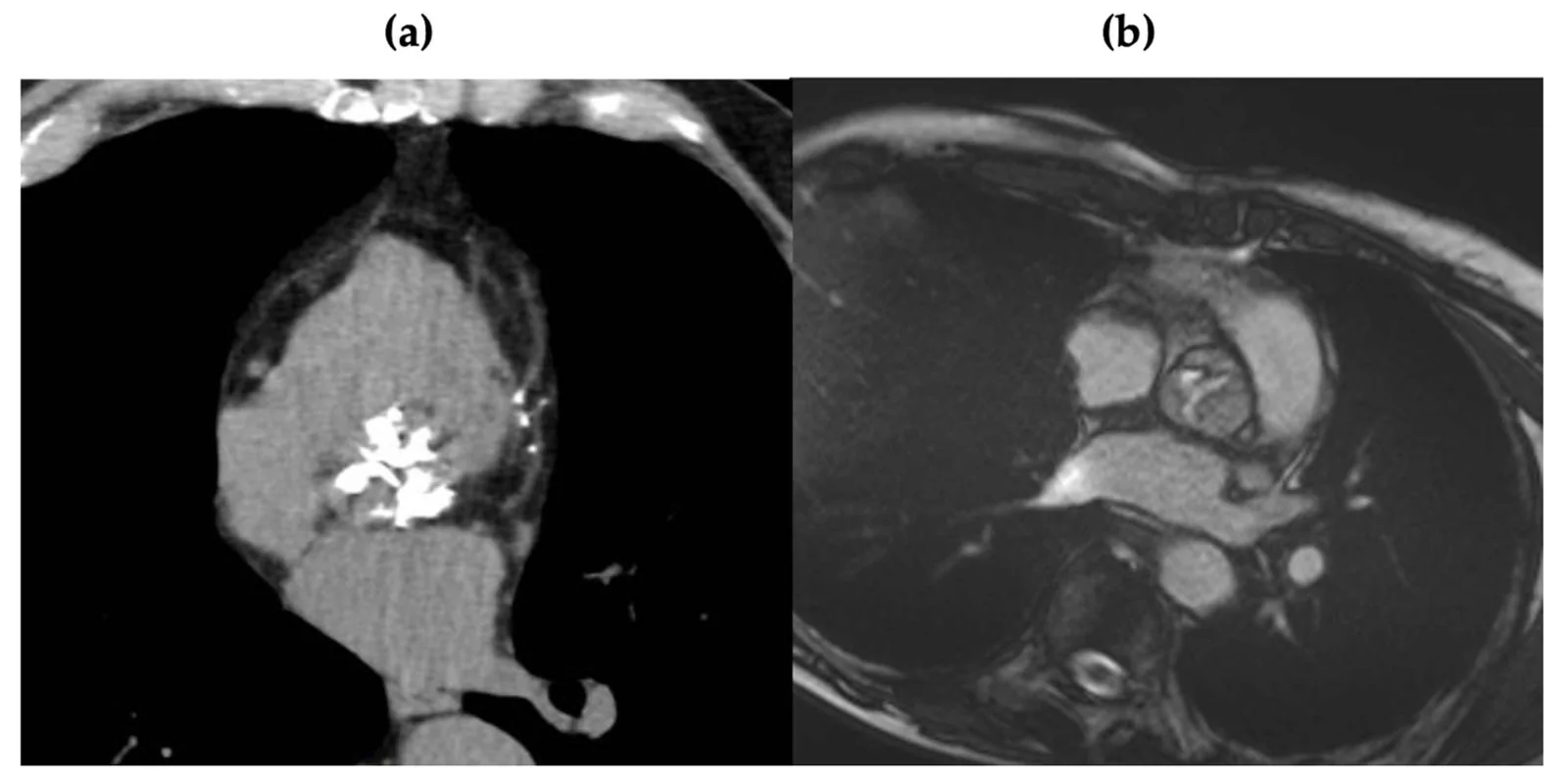
(**a**) Computed Tomography in NF-LG AS; (**b**) Cardiac Magnetic Resonance in NF-LG AS. NF-LG, normal-flow, low-gradient; AS, aortic stenosis.

**Table 1:** Multimodality Imaging in Patients with Normal-Flow Low-gradient Aortic Stenosis: Advantages and Limitations of Available Imaging Modalities.

Imaging Modality	Key Clinical Target	Advantages	Limitations
TTE	First-line screening; Phenotype stratification (NF vs. LF).	Widely available, safe, and reproducible; GLS-based subclinical LV dysfunction detection.	Operator dependency; Two-dimensional geometric assumptions of circular LVOT; Acoustic window limitations and Doppler misalignment risk.
TOE	LVOT anatomy assessment; Direct measurement of AVA planimetry	Superior spatial resolution for anatomical assessment; Direct, measurement of the LVOT area through 3D; Ruling out subaortic membranes or dynamic obstructions.	Semi-invasive procedure; Direct 2D/3D planimetry of the AVA can be technically challenging in heavily calcified valves.
MDCT	Severity confirmation via AV Calcium Score; Corrects LVOT geometry errors, hybrid AVA calculation; TAVI planning	Flow independent; Validated sex-specific cutoffs; Accurate elliptical LVOT planimetry; Reproducible and widely available; Mandatory in pre-TAVI workup; ECV-CT for ATTR-CA	No hemodynamic information; Calcium score unreliable in ATTR-CA Hybrid AVA not validated prognostically vs. Echo-AVA; Direct CT planimetry: poor correlation with gradient Contrast use and radiation exposure
CMR	AVA by PC-CMR LVOT area Myocardial fibrosis burden with tissue characterisation	Accurate LVOT area Prognostic value of LGE and ECV Detects concurrent ATTR-CA Four-dimensional flow MRI: multiplane flow reconstruction No radiation exposure	Gadolinium contrast required; Possible underestimation of peak velocities due to signal dephasing in severe AS; Limited availability PC-CMR plane-dependent
Invasive Hemodynamic Assessment	Gorlin equation for AVA; Dobutamine for contractile reserve assessment	Direct measurement of transvalvular pressure gradients; Not LVOT dependent	Gorlin equation is highly flow dependent. VO2 estimation errors (Fick method) Procedural risk as invasive procedure

Summary of the applications, advantages and limitations of the different imaging modalities available for the evaluation of NF-LG AS. AS, aortic stenosis; TTE, transthoracic echocardiography; TOE, transoesophageal echocardiography; CCT, cardiac computed tomography; CMR, cardiac magnetic resonance; GLS, global longitudinal strain; NF, normal flow; LF, low flow; LG, low gradient; LVOT, left ventricle outflow tract; AVA, aortic valve area.

## Data Availability

All data relevant to the study are included in the article.

## Abbreviations

The following abbreviations are used in this manuscript:

AS	Aortic Stenosis
AVA	Aortic Valve Area
LG-AS	Low-Gradient Aortic Stenosis
LF-LG	Low-Flow, Low-Gradient
NF-LG	Normal-Flow, Low-Gradient
TTE	Transthoracic Echocardiography
TOE	Transoesophageal Echocardiography
LVEF	Left Ventricular Ejection Fraction
LVOT	Left Ventricle Outflow Tract
SV	Stroke Volume
SVi	Indexed Stroke Volume
CMR	Cardiac Magnetic Resonance
MDCT	Multi-Detector Computed Tomography
AVR	Aortic Valve Replacement
SAVR	Surgical Aortic Valve Replacement
TAVI/TAVR	Transcatheter Aortic Valve Implantation/Replacement
LGE	Late Gadolinium Enhancement
ECV	Extracellular Volume fraction
PC-CMR	Phase-Contrast Cardiovascular Magnetic Resonance
ATTR-CA	Transthyretin Cardiac Amyloidosis
Four-dimensional flow MRI	Four-Dimensional Flow Magnetic Resonance Imaging
AVAi	Indexed Aortic Valve Area
DVI	Dimensionless Velocity Index
VTI	Velocity Time Integral
FR	Flow Rate
BSA	Body Surface Area
BMI	Body Mass Index
CW	Continuous-Wave (Doppler)
MPR	Multiplanar Reconstruction
GLS	Global Longitudinal Strain
AVC	Aortic Valve Calcification
CCTA	Coronary Computed Tomography Angiography
CAD	Coronary Artery Disease
LVEDD	Left Ventricular End-Diastolic Diameter
ECV-CT	Extracellular Volume fraction by Computed Tomography
